# Comparison of mini endoscopic combined intrarenal surgery and multitract minimally invasive percutaneous nephrolithotomy specifically for kidney staghorn stones: a single-centre experience

**DOI:** 10.1186/s12894-022-01030-7

**Published:** 2022-06-30

**Authors:** Zhi-Hao Chen, Kau-Han Lee, Wen-Hsin Tseng, Chia-Cheng Su, Kun-Lin Hsieh, Chye-Yang Lim, Steven K. Huang

**Affiliations:** Division of Urology, Department of Surgery, Chi Mei Medical Center, No. 901, Zhonghua Rd. Yongkang Dist., Tainan City, 71004 Taiwan, R.O.C.

**Keywords:** Endoscopic combined intrarenal surgery, Kidney stones, Multitract percutaneous nephrolithotomy, Postoperative pain, Staghorn stones, Stone-free rate

## Abstract

**Background:**

Staghorn stones require surgical treatment to prevent serious complications. Multitract percutaneous nephrolithotomy (PNL) causes great renal parenchymal injury and blood loss. One-stage endoscopic combined intrarenal surgery (ECIRS) entails the combined use of antegrade nephroscope and retrograde flexible ureteroscope to clear the staghorn stone, which may overcome the limitations of multitract PNL. We aimed to compare the perioperative outcomes of mini ECIRS and multitract minimally invasive PNL in staghorn stone management.

**Methods:**

This was a retrospective single-center study of patients with staghorn stones who underwent ECIRS (n = 17) or multitract minimally invasive PNL (n = 17) between January 2018 and September 2021.

**Results:**

There was a significant between-group difference with respect to Guy’s stone score. Stone size, stone burden (ECIRS group, 21.41 cm^3^; multitract minimally invasive PNL group, 20.88 cm^3^ [P = 0.94]), and degree of hydronephrosis were comparable in the two groups. There was no significant between-group difference with respect to one-step or final stone-free rates. The mean operative time was also not significantly different between the groups (ECIRS group, 140 min; multitract minimally invasive PNL group, 183 min [P = 0.63]). ECIRS was associated with significantly lesser postoperative pain (visual analog scale; ECIRS group: 0; multitract minimally invasive PNL group: 2.7 [P < 0.001]). Hemoglobin loss, postoperative blood transfusion rate, complications, and length of hospital stay were comparable in the two groups.

**Conclusion:**

Both mini ECIRS and multitract minimally invasive PNL were effective and safe for the management of renal staghorn stones with comparable operation time and stone-free rate, and complications. ECIRS was associated with less severe postoperative pain.

## Background

The reported lifetime prevalence of kidney stones in the world ranges from 5 to 20% [1–3], of which 10–20% are staghorn stones [4, 5]. In the absence of surgical treatment, staghorn stones can cause serious complications such as renal failure, infection, septic shock, and even death. The mortality rate of patients with untreated renal staghorn stone may be as high as 28% over a 10-year period [6, 7]. Surgical management of staghorn stones is inherently challenging. There are several key considerations during surgery, including use of specialized surgical techniques, stone clearance rate, risk of renal function impairment, intraoperative blood loss, and the risk of postoperative infection [8–10]. The first-line therapy for staghorn stone is percutaneous nephrolithotomy (PNL) [11, 12]. PNL entails one or more small incisions on the back of the flank area and for performing endoscopic lithotripsy operation. For complex kidney stones, a single percutaneous nephrostomy (PCN) tract may not be able to achieve stone-free status in the first attempt. Patients may require second look surgeries and bear the risk of multiple surgeries and anesthesia [13].

To achieve stone-free rate in a single operation, the surgeon may create multiple PCN tracts in different positions for different calyces. This type of operation is called multitract PNL. However, multitract PNL requires experts with skilled puncture technique and experience in performing precise puncture for stones in different renal calyces [10, 14]. Theoretically, the more the number of PCN tracts, the greater is the renal parenchymal injury and blood loss [15, 16]. In recent years, retrograde intrarenal surgery (RIRS) by using a flexible ureteroscope has gradually attained popularity for treatment of kidney stones smaller than 2 cm. The advantages of RIRS include lack of wound, less blood loss, and no damage to the renal parenchyma [17, 18]. However, there are some challenges associated with the use of RIRS for the management of stones larger than 2 cm, such as longer operative time and lower stone-free rate in comparison to PNL [19]. Therefore, endoscopic combined intrarenal surgery (ECIRS) has been developed in recent years. In the one-stage operation, a nephroscope is used to clear the calyceal and renal pelvis stones with a single PCN tract. Then, a flexible ureteroscope is retrogradely inserted from the urethra and a combination of laser lithotripsy and stone extraction basket is used to clear the remaining stones in other calyces. The stone fragments can be efficiently washed out through the channel of PCN [20, 21].

Based on the above, multitract PNL and ECIRS seem to be useful approaches for treatment of staghorn stones. However, a literature search for English-language publications revealed no clinical studies comparing these two approaches. In this study, we sought to compare the outcomes of ECIRS and multitract minimally invasive PNL for kidney staghorn stones.

## Methods

### Patient selection

We retrospectively reviewed medical records of patients who underwent ECIRS or multitract minimally invasive PNL at our medical center between January 2018 and September 2021. The inclusion criteria were patients diagnosed with partial or complete staghorn stones by computed tomography (CT). The exclusion criteria were patients with Grade I or Grade II by Guy’s stone score [22, 23], and patients diagnosed with xanthogranulomatous pyelonephritis. A total of 34 patients were identified, 17 of whom had received ECIRS, and the other 17 had received multitract minimally invasive PNL. The surgical method used is determined by the physician and patient via shared decision-making. If the patient has a ureteral stricture preventing the passage of a flexible ureteroscope, multitract minimally invasive PNL is selected. Two surgeons were involved in the surgery who worked together to perform ECIRS, and both were capable of performing multitract minimally invasive PNL independently. All patients underwent detailed preoperative evaluation including physical examination, medical history-taking, blood examination, biochemical examination, urine examination, plain abdominal radiograph (KUB), and CT. The primary outcome of the study was stone-free rate; the secondary outcomes included the operative time, blood transfusion rate, loss of hemoglobin, complications, postoperative pain control, and duration of hospital stay. Stone-free status was defined as residual kidney stones less than 4 mm in size on X-ray.

### Surgical procedure

#### Mini endoscopic combined intrarenal surgery

**Fig. 1:**
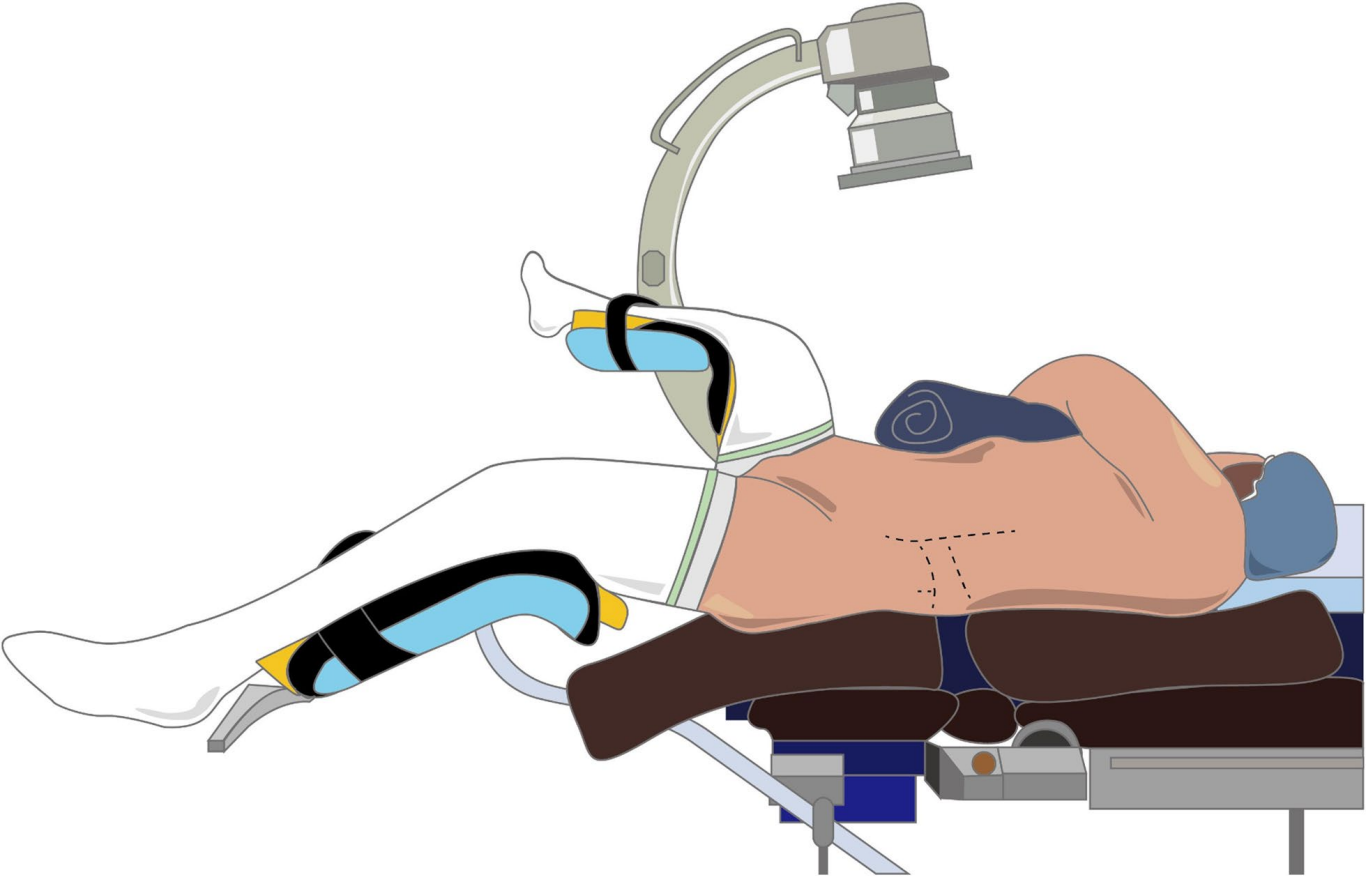
Galdakao-modified supine Valdivia (GMSV) position

**Fig. 2:**
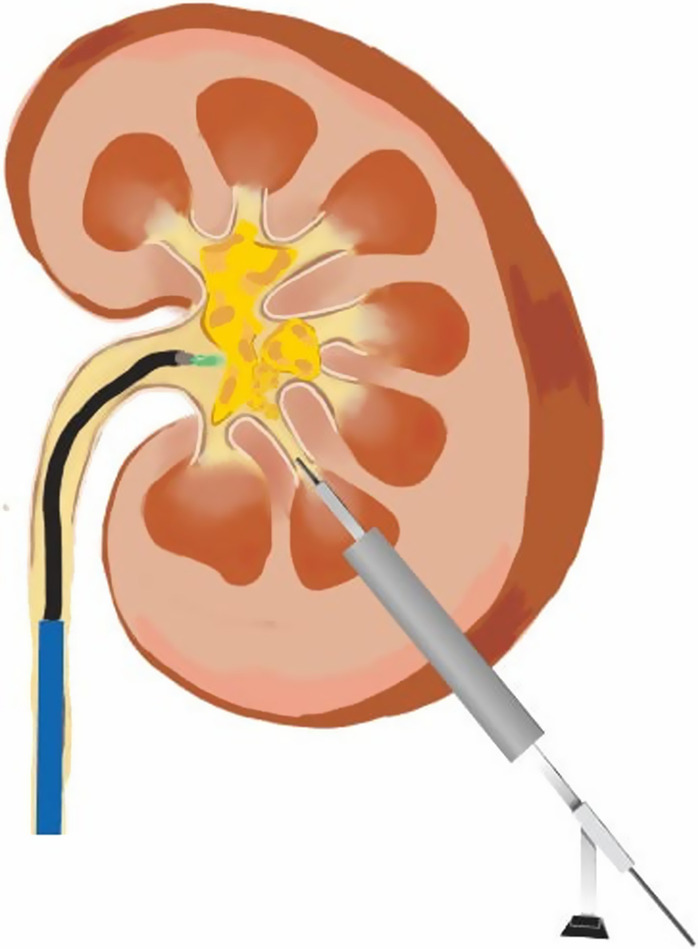
Endoscopic combined intrarenal surgery (ECIRS): a combined antegrade and retrograde approach for the treatment of renal staghorn stones

The patient was under general anesthesia and placed in the Galdakao-modified supine Valdivia (GMSV) position (Fig. 1), in which the flank on the affected side was elevated, the back of flank area was exposed, and the lower body was in the lithotomy position [24]. The CT images were checked in detail before surgery to determine the calyx to be punctured, usually the lower calyx. Intraoperatively, ultrasound and fluoroscopy were used to guide puncture. Boundaries of the safe area for renal puncture were below 12th rib, posterior axillary line, and above iliac crest. An 18-gauge needle was used under ultrasound guidance to place the guidewire into the selected calyx. Under fluoroscopic guidance, the metallic PCN dilator was sequentially expanded to 16Fr or 21Fr along the guidewire, and then the sheath of 17.5Fr or 22Fr was placed. A 12Fr nephroscope (KARL STORZ, Minimally Invasive PCNL system, medium size) with intracorporeal ballistic lithotripter (SWISS LITHOCLAST® 2) and irrigation pump was used to remove the stones in the punctured renal calyx and renal pelvis [25–27]. At the same time, a flexible ureteroscope with ureteral access sheath was retrogradely inserted from the urethra, bladder, ureter, up to the renal pelvis (Fig. 2). High-energy laser (Holmium laser Quanta System 60W) was used to break the stone in vaporization mode. A flexible ureteroscope was used to deal with the stones in the upper and middle calyx. Fragments of stones were retrieved to the PCN sheath by a stone extraction basket, and washed out by the hydrodynamic effect, known as the vacuum cleaner effect [28].

#### Multitract minimally invasive percutaneous nephrolithotomy

The surgery was performed under general anesthesia with the patient in the supine position. Preoperatively, CT images were used to determine the calyces to be punctured and to choose the adequate size of the PCN sheath. We also used ultrasound and fluoroscopy as puncture guides. An 18-gauge needle was used under ultrasound guidance to place the guidewire into the selected calyces. Under fluoroscopic guidance, the metallic PCN dilator was sequentially expanded and then the sheath of 15Fr, 17.5Fr or 22Fr was placed. A 12Fr nephroscope with intracorporeal ballistic lithotripter and irrigation pump was used to remove the stones in the punctured renal calyces and renal pelvis. Fragments of stone were washed out by the vacuum cleaner effect, and no stone extraction basket or forceps was needed.

#### Postoperative care

Postoperatively, ureteral stent was placed in all patients. Placement of the nephrostomy tube was at the discretion of the individual surgeon based on the amount of intraoperative blood loss and the number of residual stones. Postoperative pain was measured with the visual analog scale post-surgery at 6, 24, and 48 h. All the patients received 4 mg morphine as postoperative analgesic, if needed. KUB was performed immediately after the operation to determine the one-step stone-free status. Blood test, including biochemical tests, was performed the next morning. KUB and sonography were performed after 3 months to determine the final stone-free status. There were only two patients with radiolucent stones, and both were followed up with computerized tomography after surgery. Patients with clinically insignificant residual fragments were continued to be followed up for imaging results and any symptoms following surgery.

### Statistical analysis

The data of the two groups were compared using the Chi-squared test. Statistical analysis was performed using IBM® SPSS®. P values less than 0.05 were considered indicative of significant difference.

## Results

There were no significant differences between the ECIRS group and multitract minimally invasive PNL group with respect to age, sex, or body mass index. There was a significant between-group difference with respect to Guy’s stone score. Grade III of Guy’s stone score indicates staghorn stone, and Grade IV indicates complete staghorn stone. The percentage of patients with complete staghorn stone in the ECIRS group (29.4%) was significantly lower than that in the multitract minimally invasive PNL group (76.5%; P = 0.006). However, there were no significant between-group differences with respect to stone size, stone burden, or degree of hydronephrosis (Table 1).

**Table 1:** Demographic and clinical characteristics of the study population

	mini ECIRS (*n* = 17)	multitract minimally invasive PNL (*n* = 17)	*P*
Mean age ± SD	58.35 ± 14.03	58.94 ± 9.43	0.224
Sex			0.169
Male	11	7	
Female	6	10	
BMI (kg/m^2^)	26.98 ± 5.55	26.72 ± 6.31	0.832
HTN, *n* (%)	12 (70.6)	9 (52.9)	0.290
DM, *n* (%)	5 (29.4)	7 (41.2)	0.473
Previous surgery			
ESWL, *n* (%)	3 (17.6)	0 (0.0)	0.070
URS, *n* (%)	1 (5.9)	0 (0.0)	0.310
PCNL, *n* (%)	1 (5.9)	5 (29.4)	0.072
Stone side			0.730
Left	9	10	
Right	8	7	
Guy’s stone score			0.006
Grade III	12	4	
Grade IV	5	13	
Stone size (mm)	47.29 ± 15.35	66.53 ± 15.32	0.727
Stone burden (cm^3^)	21.41 ± 10.99	20.88 ± 12.22	0.936
Grade of hydronephrosis			0.362
None	7	3	
Mild	3	7	
Moderate	5	5	
Severe	2	2	

ECIRS = endoscopic combined intrarenal surgery; PNL = percutaneous nephrolithotomy; SD = standard deviation; BMI = body mass index; HTN = hypertension; DM = diabetes mellitus; ESWL = extracorporeal shockwave lithotomy; URS = ureteroscopy

The perioperative outcomes are summarized in Table 2. The one-step stone-free rate (SFR) in the ECIRS group was 64.7%, which improved to 70.6% after 1 month. The one-step SFR in the multitract minimally invasive PNL group was 58.8%, which increased to 70.6% after 3 months. There was no significant between-group difference with respect to either one-step SFR or final SFR. The mean operative time was 140 and 183 min in the ECIRS and multitract minimally invasive PNL groups, respectively; the between-group difference in this respect was not statistically significant (P = 0.63). The mean visual analog scale (VAS) score for pain at 6 h after surgery in the ECIRS group (0.0) was significantly lower than that in the multitract minimally invasive PNL group (2.7; P < 0.001). No significant differences were observed between the two groups with respect to hemoglobin loss, postoperative blood transfusion rate, complications, or length of hospital stay. After the surgery, nephrostomy tube was placed in one patient in the ECIRS group (5.9%) compared to five patients in the multitract minimally invasive PNL group (29.4%) (P = 0.072). No patients with stone-free status, including complete stone-free status and clinically insignificant residual fragments, required surgical intervention during the follow-up period.

**Table 2:** Comparison of perioperative outcomes in the two groups

	mini ECIRS (*n* = 17)	Multitract minimally invasive PCNL (*n* = 17)	*P*
Operative time ± SD	140.12 ± 63.30	183.65 ± 60.58	0.630
No. of percutaneous access tracts, *n*			< 0.001
One	17	0	
Two	0	7	
Three	0	6	
Four	0	3	
Five	0	1	
Pain VAS score			
At 6 h	0.0 ± 0.0	2.7 ± 3.1	< 0.001
At 24 h	0.0 ± 0.0	1.3 ± 2.0	< 0.001
At 48 h	0.0 ± 0.0	0.5 ± 1.4	0.003
Postoperative IV narcotics use			0.005
Yes, n (%)	0 (0.0)	8 (47.1)	
No, n (%)	17 (100.0)	9 (52.9)	
Hb drop (g/L)	1.66 ± 1.21	1.77 ± 1.01	0.068
Complications (Clavien–Dindo grade), *n* (%)			
Grade I (mild hematuria)	7 (41.2)	8 (47.1)	0.730
Grade II (fever, anemia)	4 (23.5)	3 (17.6)	0.671
Grade III	0 (0.0)	1 (5.9)	0.310
Grade IV	1 (5.9)	0 (0.0)	0.310
Blood transfusion rate, *n* (%)	1 (5.9)	3 (17.6)	0.287
One-step SFR, *n* (%)	11 (64.7)	10 (58.8)	0.724
Final SFR at 3 months, *n* (%)	12 (70.6)	12 (70.6)	1.000
Length of stay (day)	4.3 ± 3.1	3.3 ± 0.8	0.005
Nephrostomy tube, *n* (%)	1 (5.9)	5 (29.4)	0.072

ECIRS = endoscopic combined intrarenal surgery; PCNL = percutaneous nephrolithotomy; SD = standard deviation; VAS = visual analog scale; SFR = stone-free rate; IV = intravenous; Hb = hemoglobin

## Discussion

To the best of our knowledge, this is the first study in English that compares the outcomes of mini ECIRS and multitract minimally invasive percutaneous nephrolithotomy (multitract minimally invasive PNL) specifically for kidney staghorn stones. In our study, the final SFR at 3 months in both groups was 70.6%, which is comparable to the SFRs for staghorn stone reported from previous studies (49–78%) [29]. In the study by Zhong et al. (2015), ECIRS using standard PCN sheath was associated with shorter operation, higher SFR, and lesser decrease in hemoglobin than multitract PNL [30]. Zhao et al. (2021) compared ECIRS with single tract PNL for complex nephrolithiasis, which were not limited to staghorn stones. They reported higher SFR and fewer complications with ECIRS compared to single tract PNL [31]. Hamamoto et al. (2014) compared mini ECIRS, mini PNL, and conventional PNL for large kidney stones. They only used a single tract in PNL, and the kidney stones included in the study were not limited to staghorn stones. They found that mini ECIRS was superior to PNL in terms of operation time, SFR, and Hb loss [32]. Wen et al. (2016) compared mini PNL and ECIRS for partial staghorn stones. They reported a higher one-step SFR with ECIRS compared to mini PNL, while there was no significant between-group difference with respect to incidence of complications [33]. However, Wen et al. (2016) did not investigate complete staghorn stones. A recent systematic review regarding ECIRS also demonstrated that ECIRS had a better one-step SFR and fewer complications than single tract PNL in the treatment of complex stones [34].

Our study shows that use of ECIRS instead of multitract minimally invasive PNL when dealing with kidney staghorn stones can help avoid multiple tracts. We used only a single tract to clear the staghorn stones in the selected calyx and renal pelvis, and used the flexible ureteroscope with laser to remove the residual stones in distant calyces. Theoretically, since only a single tract is created, ECIRS can reduce blood loss, number of wounds, and postoperative pain compared to multitract minimally invasive PNL. In our study, postoperative decrease in hemoglobin level in the ECIRS group was lesser than that in the multitract minimally invasive PNL group, although the difference was not statistically significant. According to a meta-analysis, single tract is associated with significantly less hemoglobin loss than multitract minimally invasive PNL [19]. In our study, only one person (5.9%) in the ECIRS group required placement of a nephrostomy tube as against five patients (29.4%) in the multitract minimally invasive PNL surgery group. This is attributable to creation of only a single tract during ECIRS, which caused lesser renal parenchymal damage and blood loss; this reduced the need to drain blood after the operation. Regarding postoperative pain, none of the patients in the ECIRS group required opioid analgesics. However, nearly half of all patients in the multitract minimally invasive PNL group required morphine injection after surgery. The difference in pain is likely attributable to the lesser number of surgical incisions in the ECIRS group. Studies have shown that more the number of incisions, more severe is the postoperative pain in endoscopic surgery [35].

The operative time in ECIRS and multitract minimally invasive PNL was 140 and 183 min, respectively. Although the operative times did not significantly differ, it should be noted that staghorn stones, especially of infectious origin, must be operated within time limits. Moreno-Palacios et al. (2014) identified that surgical durations of ≥ 120 min were associated with severe complications [36]. Because multitract minimally invasive PNL provides multiple PCN routes for stone removal, it can provide better stone removal efficiency and shorten the operative time compared with ECIRS, which has only one PCN route.

The reason for using minimally invasive PNL was the small diameter of the PCN sheath with a more flexible angle for operating in the kidney during surgery, which is less likely to cause renal laceration injury. In addition, minimally invasive PNL provides a vacuum cleaner effect [28], which could wash out the stone automatically, omitting the action of clipping the stone out, thereby saving some operative time. An ECIRS systemic review by Cracco et al. (2020) included a total of 14 studies, and in all the studies except one, the insertion of a ureteral access sheath (UAS) had been reported. Although the article did not clarify the reason for requiring UAS, we consider that using an access sheath protected the flexible ureteroscope and potentially reduced the intrarenal pressure and risk of infection [37].

Some limitations of our study should be acknowledged. This was a single-center retrospective study with a small sample size. The choice of surgical approach was jointly decided by the doctor and the patient; therefore, our results may have been influenced by selection bias. Moreover, there were more patients with complete staghorn stones in the multitract minimally invasive PNL group. Although no significant difference in stone burden and size was observed between the two groups, they could still affect the study outcomes. In our cohort, we did not measure the glomerular filtration rate based on comprehensive renal function test to verify whether ECIRS is associated with lesser damage to the renal parenchyma compared with multitract minimally invasive PNL. More rigorous studies with larger number of patients are required to provide more robust comparison of the two procedures for staghorn stones. Further research is still needed to explore the cost efficacy and learning curve of ECIRS before it can be more widely used. Our study may help inform some study design considerations for future randomized controlled trials.

## Conclusion

Both ECIRS and multitract minimally invasive PNL were effective and safe for the management of renal staghorn stones. There was no significant difference between the two techniques with respect to operation time, SFR, or complications. ECIRS was associated with significantly lesser pain than multitract minimally invasive PNL.

## Data Availability

The datasets generated and/or analysed during the current study are not publicly available due to no open access to Chi Mei Medical Center Medical Records Room data base, but are available from the corresponding author on reasonable request.

## Abbreviations

CT: Computed tomography
GMSV: Galdakao-modified supine Valdivia
KUB: Plain abdominal radiography
PCN: Percutaneous nephrostomy
PNL: Percutaneous nephrolithotomy
RIRS: Recent years, retrograde intrarenal surgery
SFR: Stone-free rate
VAS: Visual analog scale
